# Towards a web-based archaeological excavation platform for smartphones: review and potentials

**DOI:** 10.1186/s40064-015-1115-3

**Published:** 2015-07-02

**Authors:** Georgios Styliaras

**Affiliations:** Department of Cultural Heritage Environment and New Technologies, University of Patras, Seferi 2, 30132 Agrinio, Greece

## Abstract

The paper conducts a review questioning the usability of a web-based platform supporting archaeological excavations and related fields, which will execute on smartphones. Based on the thorough review and comparison of related work, the basic features of such a platform are outlined. The platform should support documenting content on an underlying XML database through a content management system, producing and exchanging notes, map interaction, use of a shared whiteboard, collaboration among archaeologists etc. The architecture of the platform is presented along with two case studies supporting usual practices on an archaeological field, some primary evaluation results and future work.

## Introduction and discussion

Smartphone sales have reached outstanding numbers. According to Gartner (http://www.gartner.com), one billion smartphones were sold in 2013. Furthermore, smartphones outsold feature phones in 2013 for first time ever, whereas smartphone growth is driven by developing markets. Therefore, it is obvious that smartphone penetration is a global phenomenon and applications developed for them have potentials to reach billion users worldwide. The characteristics of smartphones, such as location awareness, touch interactivity, wireless connectivity and voice commands make them ideal for a lot application categories.

On the other hand, web technologies provide now the interface means for a variety of applications such as social media, information delivery and business operations. The evolution of browsers, their scripting languages and their capabilities have made possible the implementation of complex functions through a uniform interface. In parallel, more and more users are accustomed to interacting with a browser. Therefore, it is ordinary for software developers to exploit this infrastructure for building applications.

In this paper, the potential of using smartphones and web technologies in a platform that assists archaeological excavations and related fields is presented. The inspiration for this platform has been the involvement in working with the 3rd Ephorate of Prehistoric and Classic Antiquities in Greece for supporting the relocation of the mobile exhibits of the ephorate to a new area. In this case, which took place in 2005, a content management system has been implemented, which curators of the ephorate enriched wirelessly via an application implemented for a Pocket PC device. Visual Basic for Applications has been used for implementing the application. More than 100 fields were defined in the underlying database in order to record the characteristics and the restoration process of a single exhibit. Archaeologists could work on site and record the characteristics of mobile exhibits in the various fields with the mobile device. The application was tabbed in order to provide easy access to sibling fields. Furthermore, more and more research and actual implementations call for automation in archaeological processes such as Henson (2013), Shott (2014), REVEAL (Sanders 2011) and Stanish and Levy (2013), who enumerate the stages that this automation should follow as reviewed in the following section. During the involvement in a research project with various archaeological teams working in the Archaeological site of Stobi, the ancient city of Pompeipolis and Argos Orestikon, archaeologists working on these sites confirm this need. They currently tend to use smartphone technology but in an unorganized way.

Nowadays, with the advancement of smartphones and web technologies, such operations and the actual excavation process may be assisted even more. However, there is no complete platform that assists archaeological excavations or similar activities that has been especially designed for smartphones and takes into account modern capabilities of web technologies. In most cases, as shown in the following review section, related systems and applications are designed for desktop and use simplistic web interfaces. Smartphones are treated as another means for executing these applications. More sophisticated mobile applications have been used in dissemination of cultural content, by exploiting augmented reality, social media and multimedia playback. Concerning web technologies, there are technologically advanced applications that, once again, are used mainly in the cultural heritage dissemination process. In the excavation process, existing applications seem to exploit only the content input functions of forms. The proposed platform based on the following review supports all the actions that take place in an excavation, by exploiting as many of the smartphone capabilities as possible, through a web interface that is most suitable for a mobile device with a touch screen.

The paper is organized as follows. Firstly, through the review and comparison of related work, the rationale for the selection of characteristics for a web-based platform assisting excavations through mobile devices is presented. “Web technologies and smartphones” summarizes the features of web technologies and smartphones that are useful for such a platform. “Archaeological excavations platform” presents the platform, whereas “Case studies” presents two case studies of its usage. “Usability evaluation of the proposed platform” presents a primary evaluation based on the cognitive walkthrough method. Finally, “Conclusions/future work” concludes the paper and presents some future work.

## Review and comparison

As little work has been done for supporting archaeological excavations through information technologies and especially web technologies and mobile devices, this section presents a thorough review of the most relevant applications and systems found that support even partially the excavation process and related fields or the dissemination of cultural content as a whole and that function through web interfaces or smartphones in some parts. After the comparative presentation of similar application and systems, it is shown that there is no complete system or application for mobile devices that is intended for archaeological excavations and embraces web technologies. However, there are a lot of scattered features in these heterogeneous platforms that could be exploited in the platform proposed. In the review presented, the focus is on these characteristics.

Firstly, some related work discusses generally the need for information technologies in the archaeological excavation process. Then the review continues with some early platforms, developed prior to the smartphone era, which supported some excavation documenting operations. The use of smartphones is reviewed in projects that are applied to similar environments to excavations and in more generic cultural dissemination projects. Finally, some applications are reviewed with modern interfaces that assist in archaeological excavations.

### Theoretical background

Theoretically speaking, Henson (2013) examines how digital media and especially web technologies could be embraced effectively for disseminating archaeological content. There are examples of web sites, but the conclusion is that more needs to be done. A lot of examples covering the workflow of analysis of digital models of stone tools are presented in Shott (2014). The workflow includes Data acquisition, Digital Modelling, Analysis and Archiving. Lithic analysts employ geomorphometric methods in order to characterize, measure and analyze stone tools with newly invented mediums. The author states that once 3D models are completed to acceptable standards, finished models should be made web-accessible. Dunleavy (2014) states that Augmented Reality can exploit smartphone features such as GPS, compass, camera, object recognition and tracking in order to provide the basis for the implementation of educational applications for cultural settings. Ch’ng (2011) explores new ways for enriching the heritage tourism experience. This paper focuses on new media-enhanced exploration and learning of culture and heritage and proposes the Heritage Tourist in the Web 3.0 and Pervasive Computing Era. Web technologies and smartphone capabilities are fully embraced in the scenario presented although it concerns mainly the tourist’s experience in a heritage area.

### Early platforms

On some early platforms, which however have some sound design features, 3D Murale (Cosmas et al. 2001), contains tools for recording, reconstructing, visualizing and database querying for buildings and their parts, statues and their parts, pottery, stratigraphy, terrain geometry and texture and material texture. The tools are loosely linked together by a common database on which all users have the facility to store and access data. Web exploitation was regarded as future work. Similarly, a digital excavation data management system has been used in the “Grand Ribaud F” Estruscan deep-water wreck (Drap and Long 2001). Java has been used for formalizing and manipulating archaeological data, for digital photogrammetry and a three-dimensional model generator has been used as a navigation interface for a database.

### Smartphone applications for similar environments

On the other hand, smartphone usage is present in application such as iInteractive Tiled Display Wall (Rieko et al. 2012), where authors developed an system that allows users to explore digital heritage content with intuitive interface that operates on a smartphone. The application translates the user’s movement in the smartphone to multimedia content interaction on large displays. The application focuses on cultural content presentation in general and makes no use of web technologies. Andersen and Møbjerg (2013) describe a smartphone application designed to display prehistoric and historic finds and sites at the actual locations where events took place. An online, web-based editor tool makes it easy for museum curators to add new locations and make changes to existing ones. The application exploits augmented reality and smartphone features such as GPS to display content. On a more sophisticated application, ALERT mobile (Barreau et al. 2013) is a web-based smartphone application that exploits their GPS feature in order to allow a user to type and transmit information about vulnerable situations of coastal archaeological heritage sites to a secure server. Users may perform queries to find relevant information based on geographical and technical criteria. This application satisfies the technological criteria for smartphones and web technologies but it is not used in pure excavation work.

### Smartphone applications for cultural dissemination

Mobile devices and web technologies have been used extensively in disseminating cultural content, as in PEACH (Busetta et al. 2004), where museum visitors are provided with information about exhibits. Information may originate from the museum’s server or other remote servers and presented on mobile devices, info kiosks or large display areas. The devices sense approaching visitors and produce custom, agent produced and role-based presentations. In this case, no web technologies are engaged. Duffy et al. (2011) discuss a GPS-guided full 3D reconstruction of an ancient environment on iPhone and iPad, the Lost City of Clonmacnoise. Although smartphones are exploited, the method focuses on reconstruction and does not engage web technologies. Ardito et al. (2012) focus on the adoption of composition technologies for the creation of web-based situational, sharable interactive spaces that can engage smartphones. Authors exploited a general-purpose platform for mashup composition, and have contextualized it to respond to the needs of a community of cultural heritage operators. In Ardito et al. (2013), the same team illustrates an approach to enhance the visit experience of archeological parks. It also exploits composition technologies, End-User Development and participatory design approaches, in order to allow different users to create, use and share Personal Information Spaces. Heterogeneous content can be combined and manipulated to satisfy different information needs, thus enabling personalized visits to Cultural Heritage sites. Spaces are web-based and can be accessed by smartphones. Finally, XMAR (Brondi et al. 2012) is a framework that addresses the needs of outdoor markerless applications to be used in the field of cultural heritage. LiTe has been designed and developed on top of the XMAR framework to provide an augmented view of Piazza dei Miracoli, an artistic site in Italy. The application operates on smartphones, exploits their features such as GPS and presents a web-based interface.

### Excavation assisting applications

There are information systems assisting in excavations that make limited use of smartphone capabilities or web technologies. For example, Virtual Anthropology (Weber 2014) exploits digital technologies and brings together experts from different domains such as anthropology, biology, mathematics, computer science and engineering. Six areas constitute the term Virtual Anthropology: digitization, exposition, comparison, reconstruction, materialization and share. Among them, only the last area, share, exploits web technologies for sharing content to the public. REVEAL (Sanders 2011) is an information system that coordinates all data types used at excavations with semi-automated tools that in turn can ease the process of documenting archaeological sites, trenches and objects, recording excavation progress, researching and analyzing the collected evidence, and creating 3D models and virtual worlds. It focuses on exploiting excavation data and visualizing them. Although web-based forms are employed for entering content, there is no mention for further web exploitation or use of smartphones. Two modern approaches used in archaeological excavations are analyzed in Stanish and Levy (2013), who stress the need to engage new technologies in all stages of the archeological process, namely acquisition, analysis, curation and dissemination. Firstly, for acquisition, ArchField is proposed, a digital data collection tool by Neil G. Smith and Thomas E. Levy (http://www.antiquity.ac.uk/projgall/smith331/). ArchField is a Real-time 3D archaeological field recording system based on open-source technologies and focus on GIS capabilities. The system has been applied extensively in southern Jordan and it has been designed to meet archaeologists’ digital recording needs. The system stores all recorded content in a server-based remote database and automatically renders in the field what is being excavated. It partially uses web-based form for content editing and is designed as a desktop application for laptops. However, it can be executed on smartphones. Secondly, OpenDig (Vincent et al. 2013) is a platform for recording, editing, managing and publishing archaeological data. It comprises of three related applications, one for data entry in the field using hand-held mobile devices, another as a lightweight utility to view and edit data during an expedition and finally a full web application with complete tools for research and analysis. Web interface is active only for documentation purposes, after the actual excavation.

### Comparison

Table 1 summarizes applications and systems, previously defined, which either focus on excavations or support, even partially, web technologies or smartphones in their function. Other criteria include all common operations derived by the above analysis and the discussion with field archaeologists: the ability to gather/record data; communication/collaboration ability among researchers and workers; information transformation and analysis; content presentation and platform portability. In the comparison, Visual Anthropology is included, as it embraces web technologies and deals partially with excavations. Digital Threads across the Landscape, the Tiled Display Wall system, ALERT, the Lost City of Clonmacnoise and PEACH use extensively smartphone capabilities on related fields to archaeology. ArchField, OpenDig, REVEAL, 3D Murale and the system by Drap and Long (2001) use partially web-based technologies for assisting archaeological excavations. Finally, Situational Interactive Spaces and XMAR excel in web support and exploitation.

**Table 1:** Systems comparison

No.	Name	Smartphone	Web	Excavation	Collaborate	Gather/record data	Analyze data	Digest/present	Portability
1	Virtual Anthropology (Weber 2014)	None	Partial (through the share step)	Partial (research on already found fossils)	Partial (through the share step)	Full (through the digitize step)	Full (through the steps: expose, compare and reconstruct)	Partial (through the share step)	N/A
2	Digital Threads across the Landscape (Andersen and Møbjerg 2013)	Full (is a smartphone location based AR application designed to display prehistoric and historic finds and sites at the actual locations)	Partial (a web-based editor tool makes it easy for museum curators to add new locations)	None	Full (users contribute with new cultural heritage info)	Full (users contribute with new cultural heritage info)	Partial (add sites and modify content in-house, without a constant need for technical support)	(Full) make common cultural heritage more visible and readily available to a wider audience	(Full) Compatible with most smartphones
3	Tiled Display Wall system (Rieko et al. 2012)	Full	Partial (access supporting web content, deployment on web)	None (personal information spaces for cultural heritage dissemination)	Partial (collaborative mechanisms are favored)	None (end-user tool)	None (end-user tool)	Full (means for disseminating cultural content)	Full (deployment on different devices: web, touch screen, mobile)
4	ALERT (Barreau et al. 2013)	Full (GPS enabled ALERT mobile app)	Full (jQueryMobile, HTML5, CSS and JavaScript)	None (vulnerability assessment of coastal archaeological heritage)	Full (widen collaboration perspectives of between researchers, heritage managers and the wider community)	Full (allows the user to type and upload all the relevant information contained in the vulnerability evaluation form)	Partial (used in initial stage in the implementation of managing and research solutions)	None (used in initial stage in the implementation of managing and research solutions)	(Full) compatible with most smartphones
5	PEACH (Busetta et al. 2004)	Full (PDAs)	None	None (museum visitors are provided with information about exhibits)	Full (LoudVoice: main technique for agent coordination in ambient intelligence scenarios)	None (only for presenting information about exhibits)	None (only for presenting information about exhibits)	Full (main scopus for presenting content)	Full (presented by variety of clients (e.g., hand-held devices such PDAs, kiosks, wall screens etc.)
6	ArchField (Stanish and Levy 2013)	Partial (desktop application that may run on mobile devices)	Partial (desktop web interface)	Full (framework for in-field geographic data recording)	N/A	Full (geographic data recording, editing and reconstructions)			Partial (desktop interface running on multiple devices)
7	OpenDig (Vincent et al. 2013)	Full (iOS application)	Partial (only HTTP and semantic web exploitation)	Full (used in excavation sites)	Partial (simultaneous access to database)	Full (platform for recording, editing, managing and publishing archaeological data)			Full (desktop and smartphones)
8	REVEAL (Sanders 2011)	None	Partial (simplistic web interface)	Full (coordinates excavations with semi-automated tools that in turn can ease the process of documenting sites)	N/A	Full (document sites, trenches and objects, of recording excavation progress)	Full (research and analyze collected evidence)	Full (create 3D models and virtual worlds)	None (single piece of software running on PC)
9	3D Murale (Cosmas et al. 2001)	None	None	Full (Measure terrain, stratigraphy, buildings, building blocks, pottery, pottery sherds and statues on archaeological site)	N/A	Full (incorporates audio/video/image/text capture and creation)	Partial (indexing/integration tools)	Full (visualisation tools for viewing the outcome of database search process and for dissemination, search tools)	None
10	Digital excavation data management system (Drap and Long 2001)	None	Partial (diffusion of knowledge about the excavation via web interface)	Full (data management system for archaeological excavations)	Full (simultaneous contribution by staff members)	Full (textual, image, photogrammetry)	Full (store and manipulate data to dedicated DBMS)	Full (web-based presentation)	N/A
11	Lost City of Clonmacnoise (Duffy et al. 2011)	Full (iOS application)	None (only minor HTML contribution)	None	N/A	Full (graphical 3D models of buildings)	Full (assemble georeferenced terrain surface etc.)	Full (present reconstructed cultural heritage sites)	Partial (iOS devices)
12	Situational interactive spaces (Ardito et al. 2012)	Partial (devices with touch screens and mobiles)	Full (web-based platform)	None (oriented for dissemination)	Full (visitors create and interact with situational places)	None (oriented for dissemination)		Full (cultural heritage content dissemination)	Full (deployment on different devices web, touch screen, mobile, LIM)
13	XMAR (Brondi et al. 2012)	Full (markerless mobile augmented reality application)	Full (outcome: 3D website of Piazza dei Miracoli)	None (Only for cultural dissemination)	None (single-user application)	None (end-user application)	None (end-user application)	Full (3D real-time rendering of cultural heritage monuments)	Partial (access through Android devices and desktop computers)

In order to have a clear image on these systems, no support is denoted by “None”, partial/indirect support by “Partial”, full support by “Full” and no information available by “N/A”. As shown in Table 1, no system or application covers fully an archaeological excavation process by using web technologies and making use of smartphones.

ArchField seems to be the most complete system so far, although its interface is designed mainly for desktop computers, it focuses on GIS functions and web is engaged only for completing forms through Internet. OpenDig and REVEAL are also excavation assisting applications with modern interfaces. The interface of these projects will be evaluated in “Usability evaluation of the proposed platform”. Situational Interactive Spaces seem to better exploit modern web technologies especially regarding collaboration and mashups. XMAR and the application about Archaeological Parks also offer web-enabled virtual spaces.

## Web technologies and smartphones

In this section, based on the review, the functions that should be offered by information technologies for supporting an archaeological excavation platform are analyzed. In the same direction, web technologies and smartphones’ capabilities that should be exploited in such platform are also presented in the rest of the section.

### Information technologies in an archaeological excavation

When working on a site, firstly, archeologists should be able to make notes in every format and save them: text, drawings, audio and video. These notes should be characterized geographically and by orientation. They should be placed on maps or whiteboards and be shared with and edited by other users Similar functionality is found in 3D Murale (Cosmas et al. 2001), ArchField and OpenDig (Vincent et al. 2013) A content management system (CMS) along with a database are necessary, which will record the excavation process including information about the findings, the surrounding area and management information about the process. The notes will provide the primary content for enriching the CMS’ underlying database wirelessly. CMS with similar capabilities are present in Digital Threads across the Landscape (Andersen and Møbjerg 2013), ALERT (Barreau et al. 2013), ArchField (Stanish and Levy 2013) and OpenDig (Vincent et al. 2013). Collaboration should be favored by all means, by exchanging text/audio messages, working simultaneously on a shared whiteboard and having access to an announcements service. Shared whiteboards should support the placement of content nodes, their direct or indirect linking and grouping in various categories and their association with the database. These functions are much important especially on large-scaled archeological sites where face-to-face communication is impossible. Editing and updating information on these whiteboards should be performed without complex menus, just by voice commands, finger actions or other gestures. Similar editing functionality is found in collaboration enabling projects, Digital Threads across the Landscape (Andersen and Møbjerg 2013), ALERT (Barreau et al. 2013), PEACH (Busetta et al. 2004) and digital excavation data management system (Drap and Long 2001). Assignment of scattered content to database fields should be performed seamlessly. Primary and managerial content should be displayed through various visualizations, on historic and current timelines, maps, or grouped on whiteboards or other surfaces, allowing for archeologists to focus quickly on a portion of information or let them have a bigger picture on the information. Linking to online sources should also be supported at any time, allowing archeologists to connect, document and proceed to interpretations of new findings. Related projects that enable flexible content input are Digital Threads across the Landscape (Andersen and Møbjerg 2013), ALERT (Barreau et al. 2013), PEACH (Busetta et al. 2004) and OpenDig (Vincent et al. 2013).

### Web technologies for archeological interfaces

As already mentioned in “Review and comparison”, there are many web-based applications focusing on editing, research and analysis of cultural content, augmented reality views of monuments and Information Spaces for archaeological parks (Ardito et al. (2013). Nowadays, web technologies offer many new features that can be employed by applications assisting archaeological excavations. Digital Cultural Heritage Map (DCHM) (Mousouris and Styliaras 2013) is such an indicative application that has exploited many such features in order to implement a CMS for content experts to populate a digital map. DCHM may operate uniformly on all devices supporting HTML, without requiring installations or upgrades. Web sockets have been used for content communication, while extensive use of HTML 5 and AJAX has permitted continuous content editing and updating without requiring page reloading. Responsive design consideration has allowed the map to operate smoothly on any screen. HTML 5’s Geolocation API has been employed in order to integrate the web interface with map functionality. JavaScript is used to collect data from various components and handle errors, while OpenLayers are used to render a point on a vector layer. HTML5’s localStorage API has been used to implement “My Favorites”, a list of preferred cultural locations. The video gallery uses the HTML5 video tag that makes video embedding simpler, straightforward and plug-in independent. Editing of textual content is made via HTML5 forms. The Drag and Drop API allows to drag and upload files on the browser via event listeners. Files are uploaded using the xmlHttpRequest2 API.

During an excavation, these capabilities, such as storage APIs, continuous content communication through sockets, responsive design, multimedia tags and scripting, are necessary for on-site specialists to be able to record findings, document them and take notes, share them on maps or other areas and attach multimedia content, ranging from simple images and video to 3D models. Management and content forms for the whole process should be made available to various devices and platforms. Last but not least, social media integration and content visualization by using SVG (e.g. charts, timelines as in http://www.dipity.com) would enhance the communication and collaboration among on-site specialists and the dissemination of findings to the rest of the world. Content visualization can be based also on spatial hypermedia (Shipman et al. 2002) that extends classic hypertext and hypermedia by allowing new ways of explicit or implicit linking and relations of multimedia nodes and also by imposing direct or indirect grouping of information.

### Smartphone capabilities for archaeological excavations

Mobile devices and smartphones have proved their potential in a lot of processes regarding archaeological and cultural content in general starting from content gathering until dissemination. Before smartphones, personal digital assistants, wireless, GPS and touch capabilities have been employed in order to assist archaeologists and curators to record the location and enrich wirelessly, the properties of mobile exhibits. Now, there are smartphone applications that can transmit information about vulnerable situations of coastal archaeological heritage, provide information about exhibits and historic finds, perform excavation data recording, support media-enhanced exploration and learning of culture and heritage, support 3D reconstruction of an ancient environment and share interactive spaces.

Based on the review [especially in Rieko et al. (2012), Andersen and Møbjerg (2013) and Barreau et al. (2013)], smartphone features that can be used in the proposed platform are wireless and mobile phone connectivity for exchanging content; GPS, digital compass and map software for providing location awareness; a touch screen that is ideal for interacting with and visualizing content exploiting space; microphone/speakers and text editing software for taking audio/text notes and collaboration; camera for capturing photos, panoramas and videos for supporting augmented reality; media player for playbacking audiovisual content and animations.

## Archaeological excavations platform

In this section, based on the previous discussion and especially on the functionality of related systems and applications, a platform for supporting archaeological excavations is proposed which exploits smartphone functionality and modern web interfaces. The platform is modular, so that basic functions are always present while secondary ones are instantiated on demand.

More specifically, the platform is consisted of the following modules:

- XMLDB: A database in XML format that holds all information needed by the following modules executed on smartphones. The selection of XML for storing content ensures content integrity and portability.
- CMS: A web-based Content Management System that provides a visual interface between the database and content editors, who may load, insert, edit, update content and perform visual queries on it. All content objects are represented as nodes and can be edited by simple interface actions: touching and changing a node’s content results in respective changes in the underlying database. It also populates the rest of the modules with necessary content according to their functionality.
- Notes: It permits taking photos, recording of audio and video, writing texts and drawing sketches. Time and place are automatically stored along with notes, thanks to GPS function. All notes may appear on the Whiteboard and synchronized with the database via HTML5 storage features and xmlHttpRequest.
- Tools: SVG-based graphical tools for measuring and comparing new findings to existing ones. They support putting different objects on a climax, measuring their size or volume and comparing colors and materials. The tool may be applied to all objects that are present on the Whiteboard.
- Sources: A web-based portal that permits access to selected online sources with archaeological content, thematically organized and including historical events, geographical and geological information.
- Management: An AJAX-based graphical interface that coordinates the workflow in an archaeological excavation. It supports the definition of tasks to be completed along with roles, assignments, deadlines and critical signs. Whenever a task is updated, it appears automatically on every smartphone via the Whiteboard coupled with a notification sign. Via management, appropriate rights are also assigned to personnel for accessing or modifying content.
- Collaborate: An AJAX-based message board where messages and announcements are displayed as threads. Participating personnel may follow and contribute to discussions based on their rights. They may disregard visually a message by dragging it out of the module’s area.
- Whiteboard: A spatial area, based on SVG, HTML 5, localStorage, AJAX and web sockets, which provides a uniform access to the modules executed on smartphones. It can instantiate the previous modules for providing access and editing database content, enabling collaboration and management and providing access to tools and online sources. Moreover, it supports grouping and placing different layers of content on maps and other surfaces such as drawings and timelines. When a module is needed, users may drag it on the active area of the whiteboard, otherwise they unload it, by dropping it outside the board.
- Dissemination: This module generates automatically read-only views, multiple layers and augmented reality views of the stored content. Archaeologists can query some specific content of the database or parameterize other predefined queries in order to select content and present it graphically on a web site, e.g. the findings of a specific date, present all ancient coins found during the current excavation period etc.

**Figure 1:**
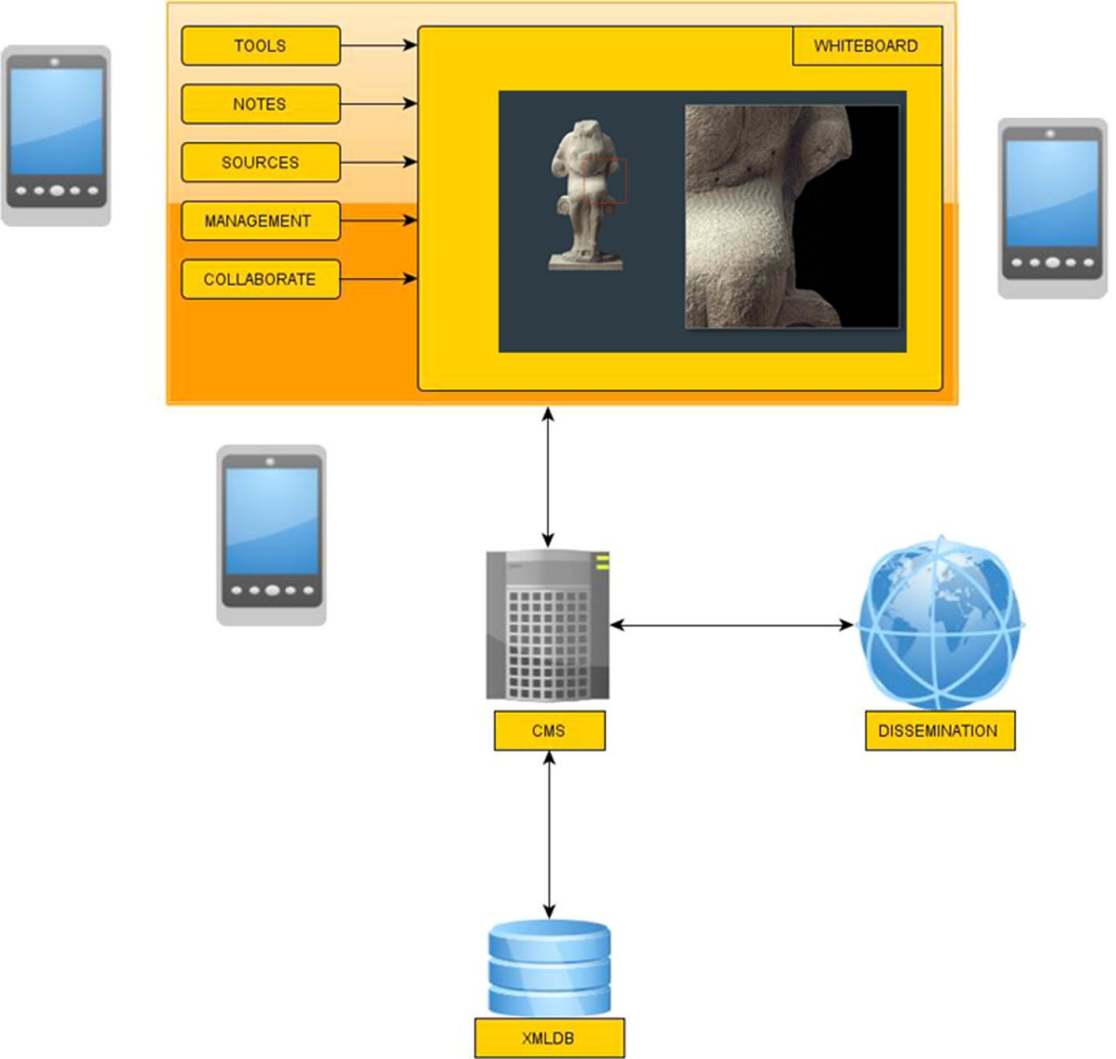
Platform architecture.

**Figure 2:**
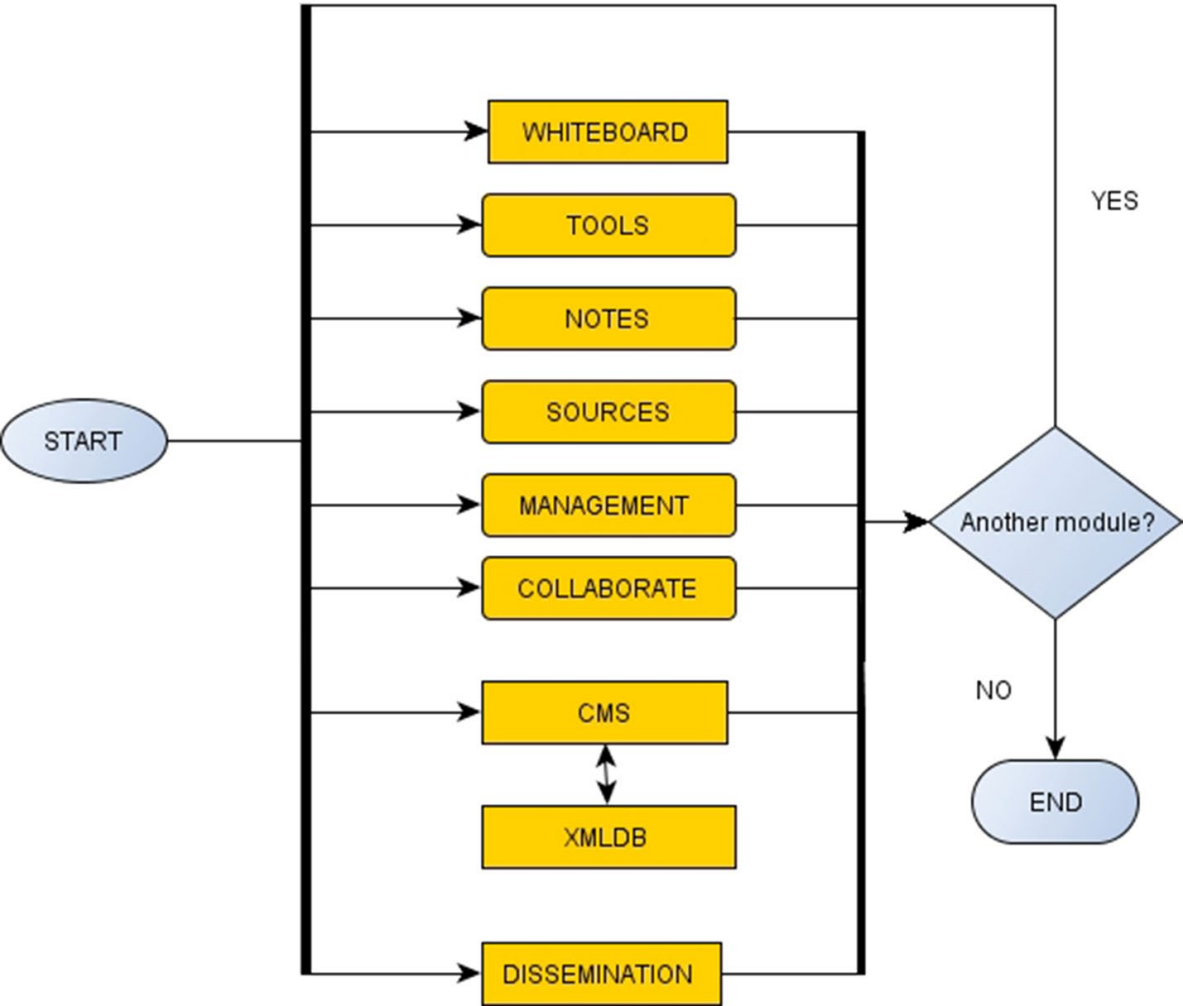
Modules’ interactivity.

CMS and Whiteboard are always present during an interaction. Through smartphones, archaeologists may configure the Dissemination module’s parameters, while the resulting web content is accessible by multiple devices. Depending on their necessity, the rest of the modules, Notes, Tools, Sources, Management and Collaborate, are instantiated on demand via the Whiteboard. Figure 1 summarizes the platform architecture and modules, whereas Figure 2 displays the interactivity among modules. Users activate needed modules in a sequential order until they are done editing and storing the needed content.

**Figure 3:**
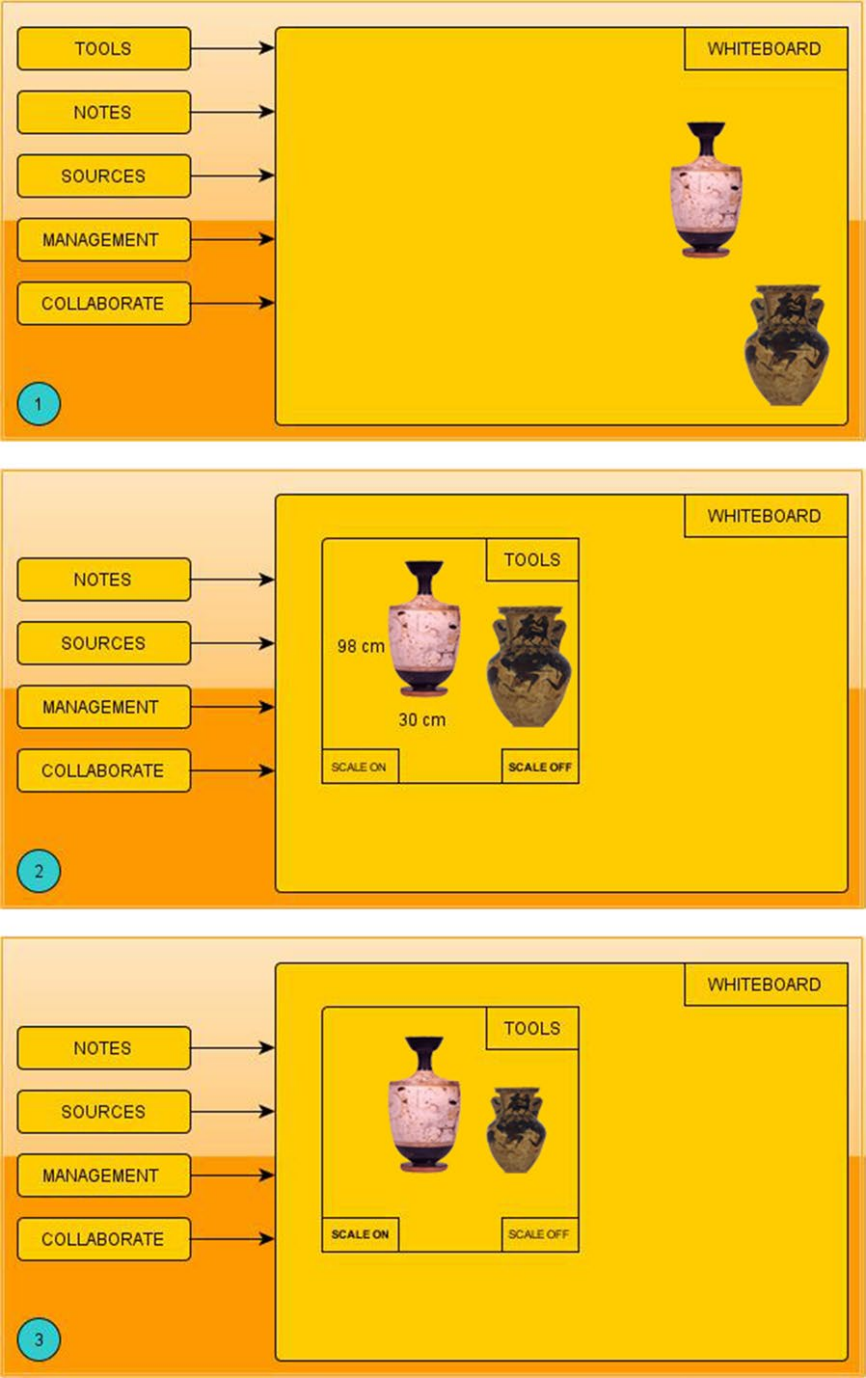
Interactivity scheme for the Tools’ module.

Upon touching on a module, this module commits an area on the Whiteboard and users may drag material on this area in order to interact with it. For example, Figure 3 shows a mockup sequence for the Tools module. There are two photographs on the Whiteboard (1). The user drags them on the Tools area and clicks on the left photograph in order to view its dimensions as they are stored in the XMLDB (2). She presses SCALE ON, this option turns bold and photographs in the Tools area are resized relatively. The user may drag the photos back to the Whiteboard or drag the Tools module outside of the Whiteboard in order to deactivate it.

Concerning hardware, a server is needed for storing XMLDB, the CMS and the web interface needed by Dissemination. Smartphones run all other modules, by retrieving and sending content to the server. An optional large interactive screen may project the whiteboard for enabling the direct cooperation among archaeologists and for presenting content to visitors via Dissemination.

Different types of personnel and public are assigned different access rights. An administrator may alter directly the database’s content, export it and disseminate it as needed. Archaeologists should be able to interact with content via the modules of the whiteboard. Public is allowed to access read-only content via Dissemination’s web site.

It is evident how smartphone features are exploited, especially regarding content organization and interaction by exploiting their surface. Similarly, web technologies play an important role for content interaction, editing and presentation. HTML 5 is used for displaying content nodes and timelines on the whiteboard and facilitates interoperability among different platforms. SVG provides graphic support for drawing nodes, events on timelines and maps and interaction. AJAX supports continuous updating of content without having to reload a page. HTML 5 Local Storage, xmlHttpRequest2 and web sockets play an intermediate role among user content interaction and permanent storage in the database. Finally, XML is used for storing content enabling content portability and exchange with content stored in other standards.

## Case studies

**Figure 4:**
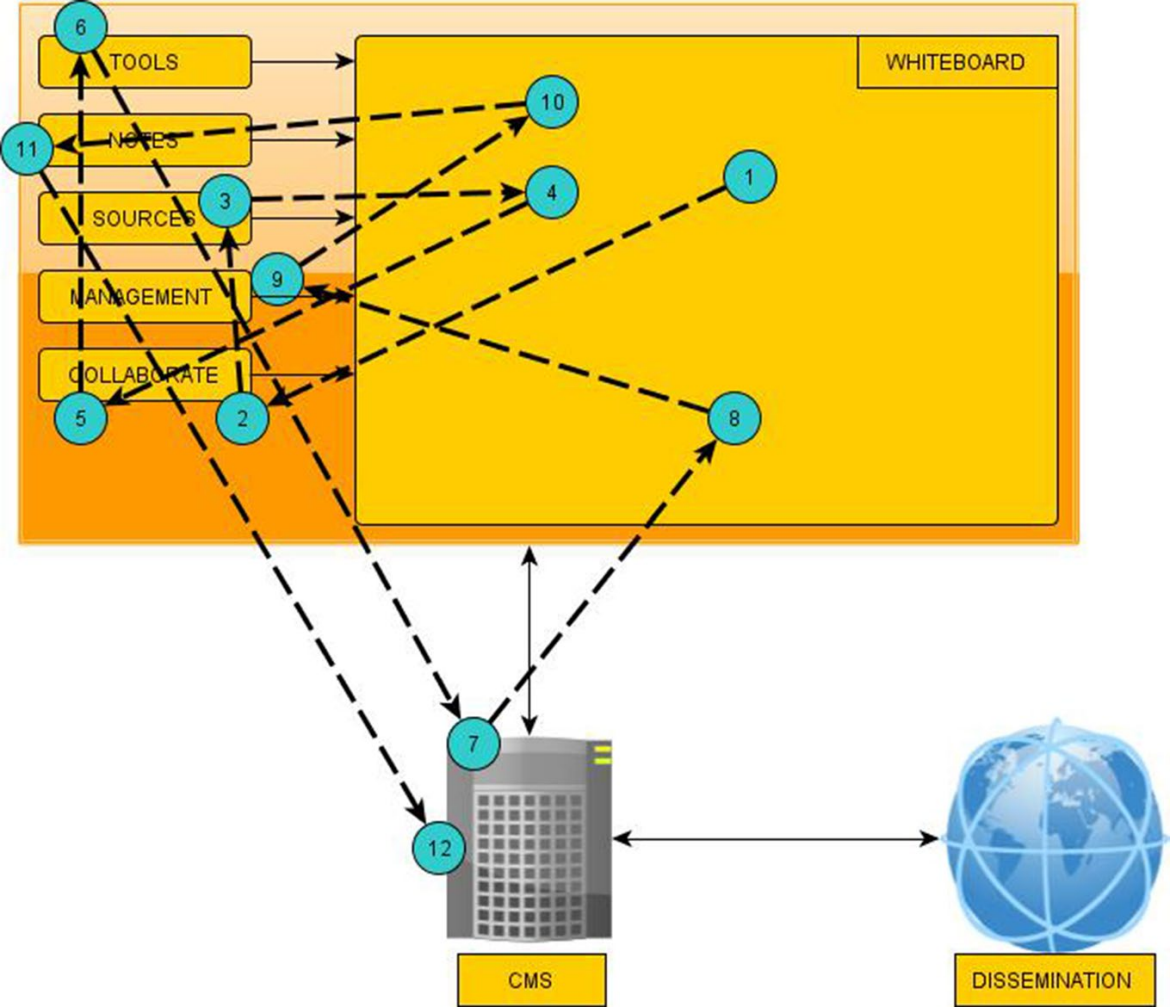
Modules’ activation sequence for Case Study 1.

**Figure 5:**
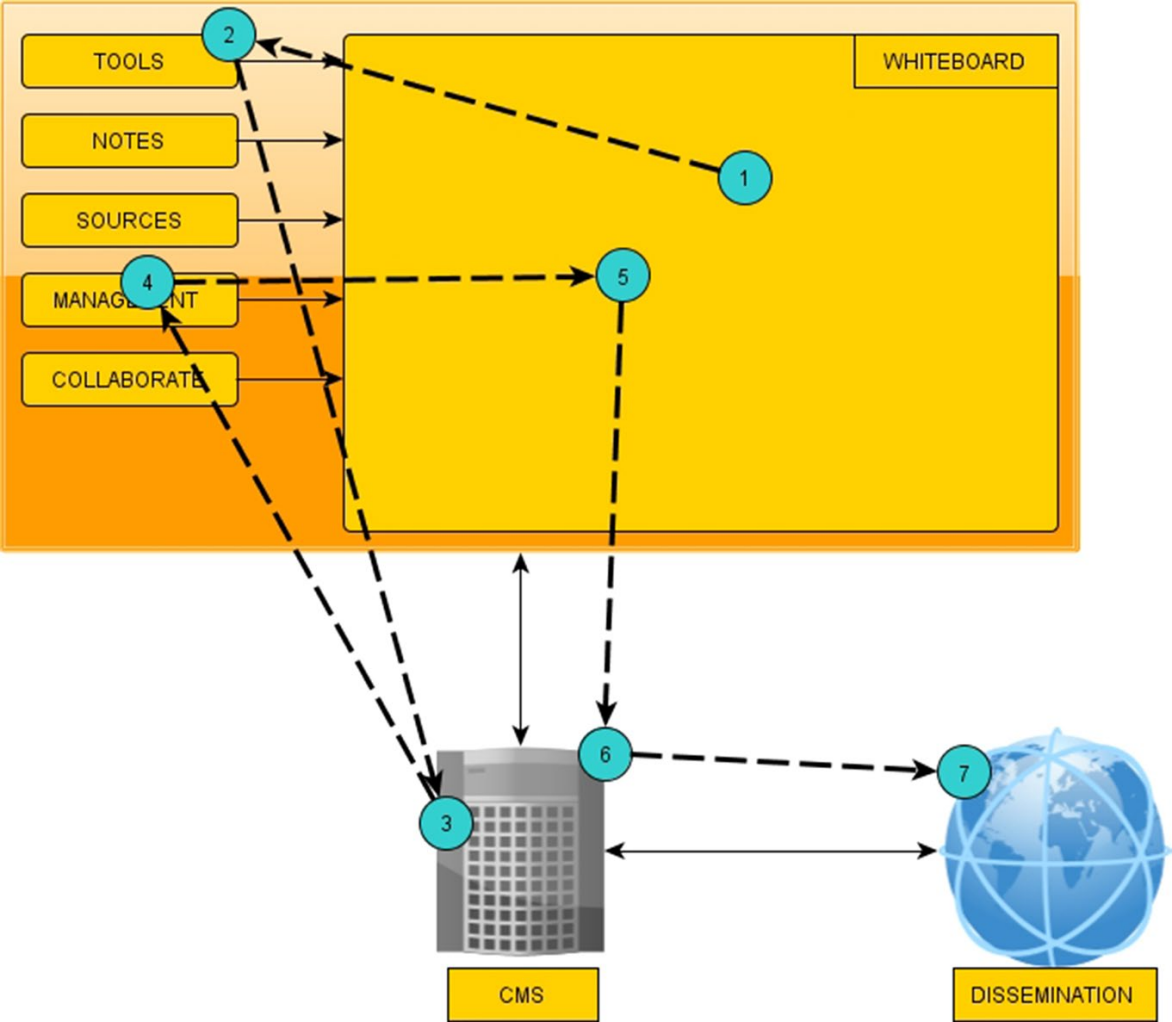
Modules’ activation sequence for Case Study 2.

Two case studies will be presented, the one showing the platform’s operation while coming across a new finding and the second about the operations taking place in the end of an excavation day. Figures 4 and 5 illustrate the modules’ activation sequence.

In a large archaeological site, there are two archaeologists, Jim and Mary, who work on different areas of the site with some workers. While working, Jim’s team finds the pieces of an ancient vase. He wants to inform Mary about the findings. He photographs the pieces, places them on the platform’s Whiteboard, groups them and names them “New vase”. The group has the coordinates of the excavation point, time and date (1). He sends a message to Mary through Collaborate (2), who reads it and joins the whiteboard in order to see the photos. She remembers a similar vase from a neighboring archaeological site, so she uses Sources (3) for identifying similar content on the other site. She drags the vase she found as similar on the Whiteboard (4). The two archaeologists continue to exchange ideas through Collaborate (5) and compare similar findings by using the climax and measure Tools (6). Mary connects to the underlying database XMLDB via CMS and after performing a suitable visual query (7), she drags on the Whiteboard some vases that should be grouped with the newly found one (8). Jim enters a new event in the excavation’s diary using Management concerning the new finding, in which all previously gathered information (time, coordinates, photographs) are linked (9). The event and respective finding are placed automatically on the excavation’s map in Whiteboard (10). They place some text using Notes (11) near the vase and save the current status in XMLDB through CMS (12), so as to visit it later in the end of this day’s work.

In the second scenario, archaeologists need to gather the material from the actions that took place during the day. They meet by displaying Whiteboard on an interactive screen (1). Jim prepares an entry for the new vase in the database. He enumerates the photographs, enters some comments and information about their storage point. He listens to the audio notes he recorded during the day and remembers to measure a fragment before archiving it by using Tools (2). After all information about the fragments are ready, they are uploaded to XMLDB through the CMS (3). Mary has to complete some digging in a specific area, so she enters a new task by using Management in the following day’s diary with the time (4), the point and the description of the work that has to be completed. They project the complete action list for tomorrow’s works and they adjust the workload by assigning technical staff where necessary. Then Mary projects the site’s map by using Whiteboard in order to have a broader image of the area and in order to discuss with Jim the areas where they should continue with their research (5). They mark these areas on the map and these are automatically saved on the database through the CMS (6). Last but not least, they select from the whiteboard this day’s most important findings through a visual query, they write a small report and publish it along with selected information on the excavation’s web site via Dissemination (7).

## Usability evaluation of the proposed platform

The need to certify the design process and prevent interface malfunctions highlights the necessity to use a formal usability evaluation method, which is suitable for a specific case. The proposed platform has been implemented only in parts, so, only its design may be evaluated. Furthermore, the platform addresses the needs of archaeologists who are supposed to fulfill certain operations based on the platform’s modules. In this direction a method is needed that evaluates the platform’s effectiveness towards achieving certain tasks. Therefore, the cognitive walkthrough method (Lewis et al. 1990), an expert-based evaluation method, has been chosen and applied for validating the platform’s functionality. It is an inspection-based method that is suitable for both formative and summative evaluation. It is executed by usability experts on the most frequently used and demanding tasks of the software application. For every task, the following questions are answered:

- Q1. Will the users try to achieve the right effect?
- Q2. Will the user notice that the correct action is available?
- Q3. Will the user associate the correct action with the effect trying to be achieved?
- Q4. If the correct action is performed, will the user see that progress is being made toward solution of the task?

If an answer to a question is negative, the usability expert should justify the answer and suggest certain improvements to solve the issue that causes the negative answer. Problems identified during the walkthrough are rated according to a discrete scale:

- 0 = no users would have problems;
- 1 = some would have problems;
- 2 = more than half would have problems;
- 3 = most users would select the wrong action.

In order to evaluate the usability of the platform, a typical task has been chosen: “Document an archaeological finding including its photograph and technical details (dimensions and excavation point)” with the following subtasks.

- T1. Gather dimensions and multimedia data about the finding collaboratively.
- T2. Measure and compare findings to existing ones.
- T3. Store and disseminate content about the finding on a database.

The cognitive walkthrough method has been applied by three usability experts, field archaeologists with experience in mobile devices and information systems for excavation content. The specialists executed the method on the proposed platform’s design (P1) and the three platforms that are oriented in excavations and have even partial support for mobile devices and web technologies, namely ArchField (P2), OpenDig (P3) and REVEAL (P4). Table 2 summarizes the findings as average values of the specialists’ rates. The proposed platform appears with less usability issues. For T3, usability experts noticed that storing content was not as straightforward as using the other visual tools. They propose integrating the functionality of the CMS in the Whiteboard, as the other modules. Concerning ArchField, although it is a powerful application for gathering geographical data and measurements, the evaluation showed that there is some lack in collaboration ability (T1), poor visual feedback in some tools (T2) and inadequate dissemination mediums, especially during fieldwork (T3). They propose that a new version is necessary designed from scratch for touch-enabled devices. OpenDig presents some minor usability problems in T1 and T2, when trying to find the right tool for the needed functionality. The specialists’ proposition is the implementation of a more straightforward visual user interface that would help improving interactivity. Finally, REVEAL presents some issues while users try to select the right tool for their tasks and in T2, they had a measuring problem. A simpler visual interface would help overcome the problems, as the specialists suggest.

**Table 2:** Cognitive walkthrough evaluation

Platforms/questions	P1: proposed platform			P2: ArchField			P3: OpenDig			P4: REVEAL		
Q1	T1: 0	T2: 0	T3: 0	T1: 1	T2: 0	T3: 0	T1: 1	T2: 1	T3: 0	T1: 2	T2: 1	T3: 1
Q2	T1: 0	T2: 0	T3: 1	T1: 1	T2: 0	T3: 2	T1: 1	T2: 1	T3: 0	T1: 0	T2: 0	T3: 0
Q3	T1: 0	T2: 0	T3: 0	T1: 0	T2: 0	T3: 0	T1: 0	T2: 0	T3: 0	T1: 0	T2: 1	T3: 0
Q4	T1: 1	T2: 0	T3: 0	T1: 0	T2: 1	T3: 0	T1: 0	T2: 0	T3: 0	T1: 0	T2: 0	T3: 0

## Conclusions/future work

This paper presented a review towards the design of a platform that supports archaeological excavations through smartphones and web technologies. As there is little previous work in this area, an extensive comparative presentation of systems and applications that are related to excavations and similar fields has revealed the features that such a platform should support. This review has shown there is no complete system that is especially designed for mobile devices and is based on web technologies for this process. Some systems focus on dissemination, others on mobile operations or are based on traditional web interfaces. The capabilities of smartphones and the evolution of web technologies call for deployment of applications with strong visual interfaces. The exploitation of space, the capability of editing content visually and the continuous update of information on a surface facilitate content management under discomfort. Field archaeologists do not have the time to click on menus and complete form items. They should be offered interfaces, closely related to the working circumstances of an archaeological site. That means few editing and clicking, less window operations, communication and collaborations capabilities, selection and relations through drag and drop and easy dissemination features. These features have been the focus of the proposed platform.

Initial discussions with field archaeologists regarding the platform have been encouraging. The primary usability evaluation of the platform has shown that users are able to perform seamlessly most common tasks. There are a lot of more powerful applications reviewed in Related Work and compared in “Usability evaluation of the proposed platform”, which are specialized in virtual reconstructions, content dissemination through augmented reality, in-field geographic data recording but lack strong and flexible visual interfaces that would make them more attractive for executing in multiple devices and useful under the specific excavation conditions.

The outcomes of the evaluation will be taken into account in the final implementation of the platform. Different parts of the platform are currently under implementation in order to be actually applied on the following excavation era. Practices of archaeologists’ work without and with the use of the platform will be compared and thoroughly evaluated. In parallel, as the platform defines a basis for distributed work with shared content and collaboration, it will be examined how it could be made to fit different research needs and be applied in related work environments and fields such as collections and arts management, exhibition designing, cultural events planning, ethnographic fieldwork in the context of folklore, historical or anthropological studies, and paleontological excavations, to name a few.
